# Enhancing Microparticle Separation Efficiency in Acoustofluidic Chips via Machine Learning and Numerical Modeling

**DOI:** 10.3390/s25206427

**Published:** 2025-10-17

**Authors:** Tamara Klymkovych, Nataliia Bokla, Wojciech Zabierowski, Dmytro Klymkovych

**Affiliations:** 1 Department of Semiconductor and Optoelectronic Devices, Lodz University of Technology, 116 Zeromskiego, 90-924 Łódź, Poland; 2 Department of Computer Design Systems, Lviv Polytechnic National University, 5 Mytropolyta Andreia, 79000 Lviv, Ukraine; 3 Department of Microelectronic and Computer Science, Lodz University of Technology, 221 Wólczańska, 90-924 Łódź, Poland

**Keywords:** acoustofluidics, microparticle separation, lab-on-a-chip, reinforcement learning, COMSOL Multiphysics, Livelink API, microfluidic simulation, machine learning, neural networks, parameter optimization

## Abstract

An integrated approach for enhancing microparticle separation efficiency in acoustofluidic lab-on-a-chip systems is presented, combining numerical modeling in COMSOL 6.2 Multiphysics^®^ with reinforcement learning techniques implemented in Python 3.10.14. The proposed method addresses the limitations of traditional parameter tuning, which is time-consuming and computationally intensive. A simulation framework based on LiveLink™ for COMSOL–Python integration enables the automatic generation, execution, and evaluation of particle separation scenarios. Reinforcement learning algorithms, trained on both successful and failed experiments, are employed to optimize control parameters such as flow velocity and acoustic frequency. Experimental data from over 100 numerical simulations were used to train a neural network, which demonstrated the ability to accurately predict and improve sorting efficiency. The results confirm that incorporating failed outcomes into the reward structure significantly improves learning convergence and model accuracy. This work contributes to the development of intelligent microfluidic systems capable of autonomous adaptation and optimization for biomedical and analytical applications, such as label-free separation of microplastics from biological fluids, selective sorting of soot and ash particles for environmental monitoring, and high-precision manipulation of cells or extracellular vesicles for diagnostic assays.

## 1. Introduction

Acoustofluidics—an interdisciplinary field combining acoustics and microfluidics—has shown remarkable growth in the past decade due to its biocompatibility, label-free nature, and non-contact manipulation capabilities [1,2,3]. Carefully tuned acoustic fields allow for precise microparticle separation, enabling point-of-care diagnostic tools that outperform traditional separation techniques [4,5,6,7,8]. Recent progress in surface acoustic wave (SAW) technologies has further improved nanoparticle manipulation and targeted drug delivery [9,10], establishing acoustofluidics as a critical tool in biomedical and analytical research [11].

The task of separating microparticles within a mixture is multiparametric. Analytically, it can be described by a system of second-order partial differential equations. No analytical solution exists for such a system, thus numerical methods are traditionally employed. While modeling significantly simplifies the design process, the large number of parameters necessitates extensive and time-consuming simulation experiments [12,13]. The development of standing surface acoustic wave (SSAW) and traveling surface acoustic wave (TSAW) technologies has enabled high precision in cell manipulation [14,15].

The design of microfluidic/acoustofluidic devices (lab-on-a-chip, or lab-chips) is a complex interdisciplinary task requiring knowledge of solid mechanics, hydrodynamics, acoustics, chemistry, materials engineering, microelectronics, and more. Therefore, the intellectualization of the lab-chip design process, based on the application of artificial intelligence (AI) in modern Computer-Aided Design (CAD) systems, is extremely important. Recent advancements in microfluidics include the development of organ-on-a-chip systems for personalized medicine [16,17].

Machine learning (ML) has revolutionized data analytics, moving the field past traditional statistical methods that relied on explicit inference. ML’s power comes from its ability to learn directly from data to identify hidden patterns and generate predictions, requiring very few assumptions about the underlying mechanisms. Crucially, this transformation was enabled by significant boosts in computing power stemming from ongoing reductions in the size and cost of electronic components (semiconductor advancements). Traditional statistical approaches often rely on a priori assumptions, such as fixed error distributions or predefined probabilistic models, and frequently depend on human intervention and intuitional of which can introduce bias and reduce reproducibility [18]. ML algorithms can process large datasets, recognize hidden patterns, and make predictions, thus proving essential in solving complex problems across various disciplines [19,20]. Their effectiveness in biomedical, engineering, and scientific applications has been widely demonstrated [21,22,23,24]. In acoustofluidics, ML can accelerate optimization by predicting particle behavior and control parameters based on simulation or experimental data, eliminating costly trial-and-error cycles. Within the EC’s initiative of “Measures aiming to reduce the presence in the environment of unintentionally released microplastics from tires, textiles and plastic pellets”, knowledge gaps about micro- and nanoplastics (MNPs) in environmental, drinking and bottled water have been acknowledged for correction, including those about: risks and occurrence; harmonized methods for sampling, processing, data analysis, and reporting. For example, the initiative Cost action will plan to provide significant about this problem [ICPLASTIC CA23131] [25]

Reinforcement learning (RL), a branch of ML particularly suited for dynamic system control, has shown promise in learning optimal sorting strategies in microfluidic systems via trial-and-error interactions [26,27,28,29,30]. Deep learning techniques have also been applied in droplet generation and real-time microchannel flow control [31,32]. Moreover, ML methods have been utilized in a broad spectrum of biomedical tasks, including early disease detection, treatment optimization, immunodiagnostics, and biosensing [33,34,35,36,37,38,39].

Microfluidic platforms enable high-throughput experimentation, generating large volumes of structured data ideal for ML training [40,41]. In particular, ML models can be trained to predict flow behavior, optimize channel geometries, and extract sorting metrics from image data using frameworks such as TensorFlow and OpenCV [42]. Applications include real-time analysis and feedback control in systems integrating sensors and actuators.

A growing body of research demonstrates the integration of ML into microfluidic chip design for tasks such as biomarker detection, cell classification, and therapeutic targeting [43,44,45,46,47,48,49,50,51,52]. Approaches include supervised learning, deep learning, and transfer learning, depending on data availability and task complexity [53,54,55]. As acoustofluidics expands toward nanoparticle delivery and precision bioparticle manipulation [53,54], AI-powered methods offer a scalable and intelligent solution for controlling particle behavior within lab-chips.

## 2. Materials and Methods

A model of a lab-chip for particle sorting was constructed using the interactive environment Comsol Multiphysics [55,56,57,58]. The resulting model allowed for the separation of two types of particles, differing in size and density, with efficiencies up to 100%. However, the process of adjusting the model and selecting appropriate input parameters proved to be too labor-intensive. Specifically, over 100 long-duration experiments had to be conducted and analyzed to determine the fluid velocities in the input channels to achieve 100% separation for just one set of “large”–”small” particles. Consequently, the decision was made to leverage Artificial Intelligence (AI) to eliminate manual selection of input parameters and further minimize errors in microfluidic calculations.

Integration of AI algorithms implemented in Python with COMSOL Multiphysics is achieved through the use of a specialized Application Programming Interface (API). This interface enables the creation of custom scripts that automate various modeling processes. The MPh library in Python provides functionality for interacting with the COMSOL API, allowing direct access to and manipulation of model parameters within COMSOL Multiphysics.

To extend functionality, the following ready-to-use libraries are available.

- Numpy: The primary library for working with multi-dimensional arrays and performing numerical computations in Python. It is used for mathematical operations in data preparation and modeling.
- Pandas: A tool for working with tabular data (DataFrames) and analyzing large datasets. It is employed for preparing and analyzing data from numerical modeling in COMSOL.
- Scikit-learn: A popular machine learning library for implementing fundamental algorithms. It is used to create basic AI models for predicting the behavior of systems in lab-on-a-chip devices.
- XGBoost: A fast and efficient gradient boosting algorithm suitable for working with large datasets. It is used to build accurate models for predicting lab-on-a-chip parameters.
- TensorFlow-GPU: A library for creating and training deep learning models that utilize GPUs to accelerate computations. It is used to develop deep learning models that predict the behavior of fluids or other physical processes within lab-on-a-chip devices.

### Design of the Lab-Chip Structure

The acoustofluidic structure fabrication process was carried out using the laboratory facilities of the Department of Semiconductor and Optoelectronic Devices at the Lodz University of Technology. Among the wide range of available technological and measurement equipment, the following were used in particular: a RAYLASE SS-II-15[Y] laser processing station and an AUREL C920 screen printer for IDT on LiNbO3 (SRC «Electron-Carat», Lviv, Ukraine) wafers using silver paste screen printing. For a real experiment bought Polyethylene microspheres (Cospheric LLC, Goleta, CA, USA).

**Figure 1:**
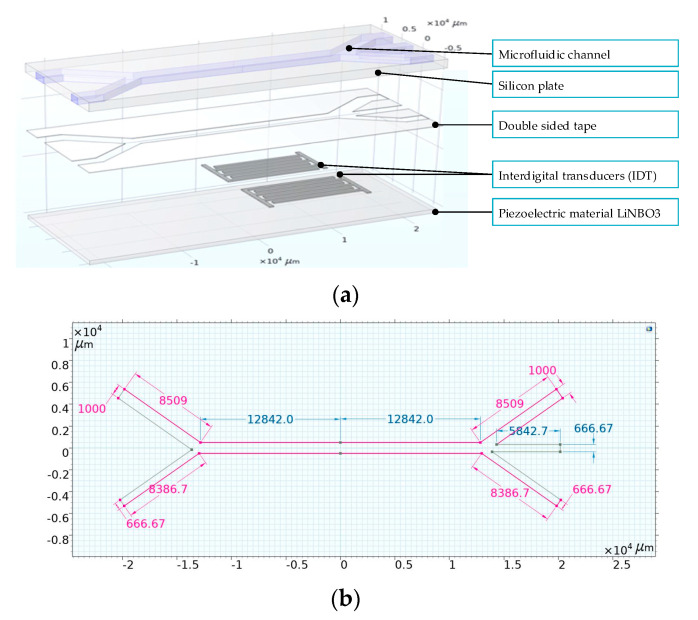
Design of the Lab-Chip structure: (**a**)—general view, (**b**)—dimensions of microchannels.

The Lab-Chip structure was designed using COMSOL 6.2 Multiphysics software. The device’s architecture for particle sorting comprises a lithium niobate (LiNbO_3_) piezoelectric substrate integrated with interdigital transducers (IDTs), and a silicon wafer containing a microfluidic channel (Figure 1). The detailed dimensions of the microchannels are presented in Figure 1b. The thickness of the electrodes is 0.002 mm, the width of the fingers is 0.5 mm, and the distance between the fingers is 0.25 mm. The thickness of the lithium niobate substrate is 0.7 mm, and the thickness of the silicon wafer is 1 mm. These components are connected together using double-sided adhesive tape, the thickness of which is 0.1 mm. These components are bonded together using double-sided adhesive tape. Comprehensive analysis of this model necessitates the integration of various Multiphysics phenomena, such as thermoviscous acoustics, particle tracking within fluid flow, creeping flow dynamics, and the intricate Multiphysics of fluid-particle interactions.

When modeling particle motion in a liquid, it is assumed that the influence of the particles on the flow field is negligible. First, the flow field is calculated, and then, as an analysis step, the particle motion is calculated. The density and radius of the first particle are 1050 kg/m^3^ and 0.5 μm, the density and radius of the second particle are 5250 kg/m^3^ and 5.0 μm, respectively. The particle mixture enters through the inlet 1 microchannel, and pure water flows into inlet 2. In the absence of acoustic force, they move in laminar flow without separating along the horizontal channel and exit through the outlet 3.

**Figure 2:**
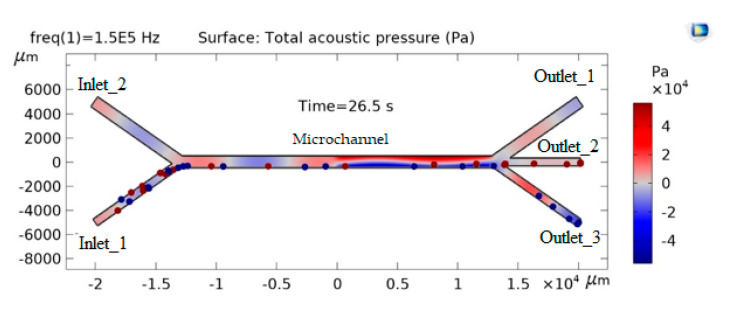
Distribution of particles in microchannels under the influence of applied acoustic force.

When an acoustic force is applied, an acoustic pressure field is applied to the velocity field. The acoustic force acts on particles of different densities and sizes in different ways (Figure 2). Heavier particles are placed at the nodes of the acoustic wave, and lighter particles are located under the wall.

In the first half of the horizontal channel, particles move under the influence of the fluid flow along the lower wall of the channel. In the second half of the channel, when the acoustic force begins to act on the particles, the heavier ones rise and move along the zero-pressure zone, while the smaller ones continue to move along the lower wall. Under the influence of the current, the heavier particles are transferred to the middle branch of the channel, and the lighter particles to the lower branch of the channel. In this way, the separation of particles occurs, the efficiency of which reaches 100%. Thus, the constructed model allows for the study of the separation process of particles suspended in a liquid and the selection of optimal parameters of the acoustofluid Lab chip.

As shown by the model studies, the efficiency of microparticle separation is influenced by the channel geometry, acoustic pressure force, liquid velocity at the inlets, density and size of particles. It is planned to carry out research on the designed prototype of a laboratory chip for sorting microparticles using the Micronit assembly kit. This device consists of two independent precision pumps that provide fluid supply with flow control in the range from 0.1 to 10 mL/s, as well as tubes and mounting accessories that significantly facilitate the creation of tight microconnections between tubes and channels. However, in such a case, the geometry of the designed microfluidic structure in terms of its total thickness, external dimensions and the arrangement of holes for connection with microchannels must be strictly adapted to the dimensions of the Micronit holder. Therefore, the range of changes in the geometric parameters of the model is limited. Since the diameter of the holes in the Micronit holder is 1.0 mm, the width of the basic channel was assumed to be 1 mm, although the model was tested in a wider range of channel widths—from 0.5 mm to 3 mm.

The first task was to determine the value of the acoustic pressure force and fluid flow velocity at the inlets, as well as to predict the percentage separation of microparticles with a known channel width and specific particle characteristics. The second task is to provide a prediction of the separation accuracy of microparticles with different physical properties and specific channel parameters.

First of all, it is necessary to determine the oscillation frequency in order to obtain a standing wave in the channel and a sufficient sound pressure force. To create a standing wave, it is necessary that the width of the channel is a multiple of half the wavelength, and then

(1) $$f=\frac{c_{0}}{\lambda },\;\;h=n\frac{\lambda }{2}\;=>\;f=n\frac{c_{0}}{2h}$$

where ***f*** is the oscillation frequency, ***c*_0_** is the velocity of sound in water, ***h*** is the width of the channel, ***λ*** is the wavelength, ***n*** = 1, 2, ….

**Figure 3:**
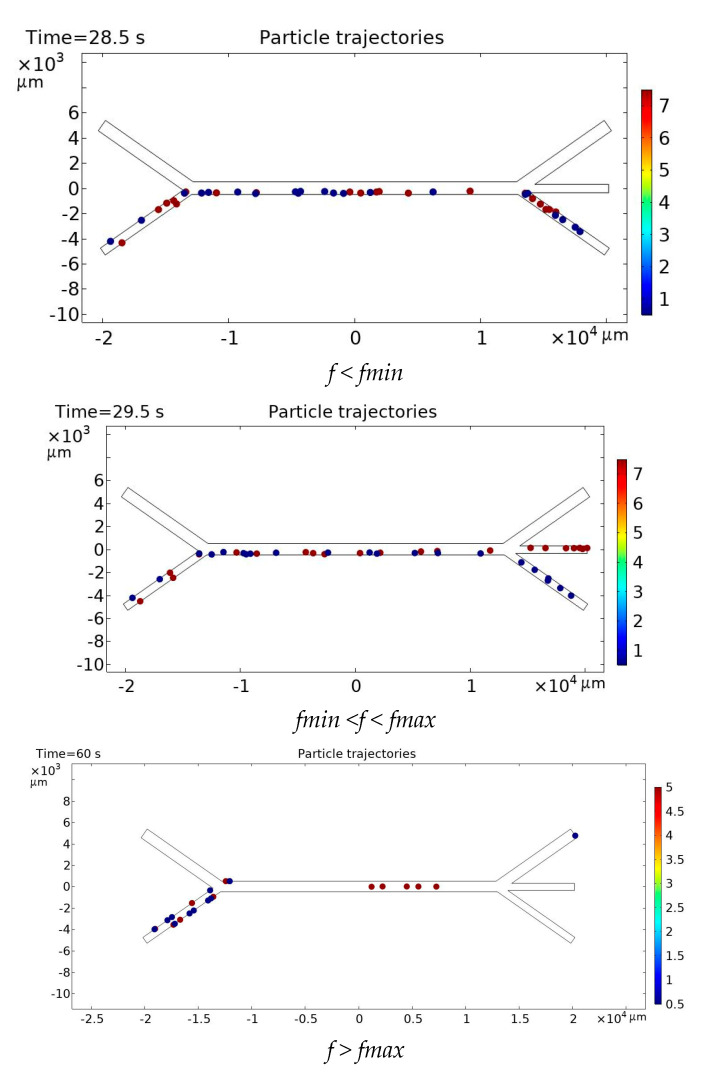
Effect of acoustic excitation frequency on particle distribution in the microchannel.

Of course, with an increase in n, the frequency will increase. However, it turns out that if the frequency is less than the critical minimum value *f_min_*, the particles will not separate, and if the critical maximum value *f_max_* is exceeded, the particles in the model will stop (Figure 3).

Using the developed microparticle separation model, empirical regression equations were constructed to determine the frequency ranges corresponding to the onset (*f_min_*) and maximum efficiency (*f_max_*) of particle separation:

(2) $$f_{min}=\mathrm{228000}-\mathrm{2600}r$$

(3) $$f_{max}=\mathrm{111.5532}-\mathrm{0.1516}r\;,$$

where ***r*** is the radius of the microparticle (in µm). These relationships were obtained by regression analysis of the simulation results and are not directly derived from the classical acoustofluidic radiation force equation but rather represent an empirical approximation of the frequency–radius dependence observed in the numerical model. The average approximation error of Equation (2) is 1.71%, and of Equation (3) is 6.47%. Both regression models are statistically significant (i.e., their coefficients are jointly reliable at *p* < 0.05). The next step is to select the flow rates at the inputs of the laboratory chip. The velocities were determined for a base channel width of 1.0 mm. At this channel width, the Micronite pump provides a minimum water delivery rate of 0.3 mm/s to the lower channel and a maximum water delivery rate of 40.0 mm/s to the upper channel. Microparticles with a density of ***ρ***_1_ = 1050 kg/m^3^ and a radius of ***r***_1_ = 0.5 μm were taken as reference points. The density of the second microparticle varied from ***ρ*_2_** = **5*ρ*_1_** to ***ρ*_2_** *=* **10*ρ*_1_**, the radius from ***r*_2_** = 5***r*_1_** to ***r*_2_** = 20***r*_1_**. In total, more than 100 tests were performed with different values of different velocity. A selection of velocity options with 100% separation of the two particle types is shown in Table 1.

**Figure 4:**
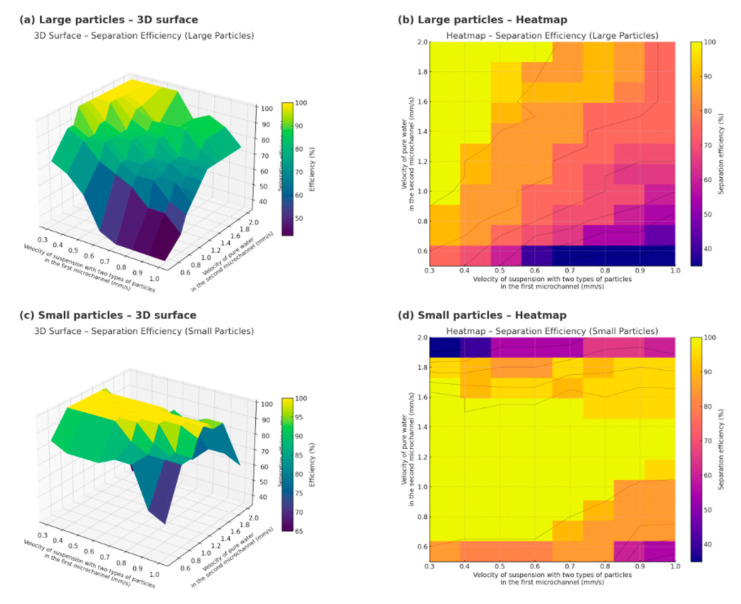
Comparison of microparticle separation efficiency for large and small particles under varying inlet flow velocities in a branched acoustofluidic microchannel: (**a**)—3D surface plot for large particles; (**b**)—heatmap for large particles; (**c**)—3D surface plot for small particles; (**d**)—heatmap for small particles.

As shown in Table 1, for particles whose radius differ by a factor of 10 or more, several velocity combinations can be selected; in fact, the condition for separation is that the velocity of pure water should exceed that of the suspension by several times (the greater the difference in radius, the smaller the required difference in velocity). However, for particles with similar radius, the separation task becomes more complex (see Figure 4).

The figure shows the simulation results of the efficiency of microparticle separation in a microfluidic lab-on-a-chip under the influence of an acoustic field at different flow rates of fluids supplied to two inlet microchannels: (a)—3D surface of separation efficiency for large particles as a function of the particle suspension velocity (X) and the pure water velocity (Y); (b)—corresponding heatmap for large particles, visualizing the regions of maximum efficiency; (c)—3D surface of efficiency for small particles under similar conditions; (d)—heatmap for small particles.

The axis labels are given with units in mm/s to emphasize the practical use of flow velocities in microchannels during experiments. The bright yellow zones on the graphs correspond to the regions of maximum separation efficiency.

To construct this figure, 88 simulation cycles were performed at different inlet velocities of the fluid in the first and second microchannels for microparticles with the radius R_1_ = 0.5 × 10^−6^ m and R_2_ = 5 × 10^−6^ m and densities ***ρ*_1_** = 1050 kg/m^3^ and ***ρ*_2_** = 2250 kg/m^3^.

As shown in Figure 4, larger particles require the velocity in the second inlet microchannel to be at least 1 mm/s for effective separation, and the ratio of the fluid velocity in the first microchannel to that in the second should be no less than 1:3. For smaller particles, there exists an optimal velocity range in the second inlet microchannel of 0.9–1.4 mm/s. Moreover, the greater the deviation from the optimal velocity of 1.2 mm/s, the lower the fluid velocity in the first inlet microchannel should be.

Considering the geometry of the microchannel and the reference size of the microparticles, a separation accuracy of 100% was achieved for particles whose density differed by a factor of five and radius by a factor of seven. However, when the channel width was set to 2.0 mm, particles with radius differing by a factor of three were successfully separated. Since selecting the liquid inlet velocities for separating particles with different physical properties is challenging, the velocity in the channel carrying water with suspended particles was first determined, and then, using this value, the liquid velocity in the second inlet channel supplying pure water was adjusted accordingly. Of the 105 experiments conducted on particles with different physical properties and different inlet velocities 16 variants were selected for which the separation efficiency was greater than 95% and multifactorial analysis was applied on their basis.

(4) $$V_{1}=\mathrm{0.0425}+0\rho_{1}-\mathrm{0.0005}\rho_{2}+\mathrm{9.004}E+\mathrm{16}r_{1}+0r_{2}$$

(5) $$V_{2}=-\mathrm{0.0793}+\mathrm{5.4737}V_{1}+0\rho_{1}-\mathrm{0.000596}\rho_{2}+\mathrm{1.783}E+\mathrm{16}r_{1}+0r_{2}$$

Estimation of the significance of the multiple regression equation carried out by testing the hypothesis of equality of the zero coefficient of determination calculated from the general population data using Fisher’s F test showed that the resulting regression equations are statistically unreliable. This means that they cannot be used for prediction and further analysis.

Thus, taking into account the channel geometry and the physical properties of the particles (their size and density), it is possible to select the frequency to generate acoustic pressure in the microchannel and determine the initial velocities that allow sorting microparticles with 100% accuracy. Or, based on empirical relationships and model experiments on the channel geometry and input conditions, to make recommendations on the possibility of particle separation. However, these tasks are time-consuming and require a large number of experiments.

Recently, along with physicochemical methods of solving microfluidic problems, much attention has been paid to computer approaches, in particular, data processing and device control using machine learning. Compared with conventional feedback control, which requires the development of unique mathematical formulas, ML can solve problems simply by collecting experimental results, and reinforcement learning (RL), a type of ML, is suitable for exploring optimal control conditions. While RL can pose challenges in providing the level of accuracy that can be achieved by mathematically describing phenomena, it reduces the workload and time of engineers by eliminating the need for complex theoretical constructions.

## 3. Computational Workflow for COMSOL-Based RL Using Python and LiveLink

### 3.1. COMSOL and Python Integration via LiveLink

In a reinforcement learning (RL) framework for microfluidic experiments, it is crucial to seamlessly integrate a high-fidelity physics simulator with the learning algorithm. COMSOL Multiphysics^®^ serves as a virtual experimentation platform, accurately simulating microparticle behavior under various flow and acoustic conditions, while a Python-based RL agent optimizes the control parameters. By coupling COMSOL with Python, the system can automatically iterate through experiments: the RL agent proposes new input values, COMSOL simulates the microfluidic outcome, and the results are fed back into the algorithm. This closed-loop integration accelerates the search for optimal microfluidic control strategies by enabling high-throughput virtual experiments and reducing the need for manual intervention.

COMSOL Multiphysics provides a programmatic interface (API) that enables external control of models and simulations mph.readthedocs.io. The capability is implemented through COMSOL’s LiveLink technology and the open-source MPh Python library, which provides a bridge between Python and COMSOL’s Java API (mph.readthedocs.io). Using this interface, a Python script can launch the COMSOL engine, load a prepared mph model file, programmatically adjust model parameters, execute the solver, and retrieve the simulation results for analysis mph.readthedocs.io. This two-way communication ensures that the RL algorithm in Python can automatically update the simulation inputs and obtain output data at each iteration without manual intervention.

**Figure 5:**
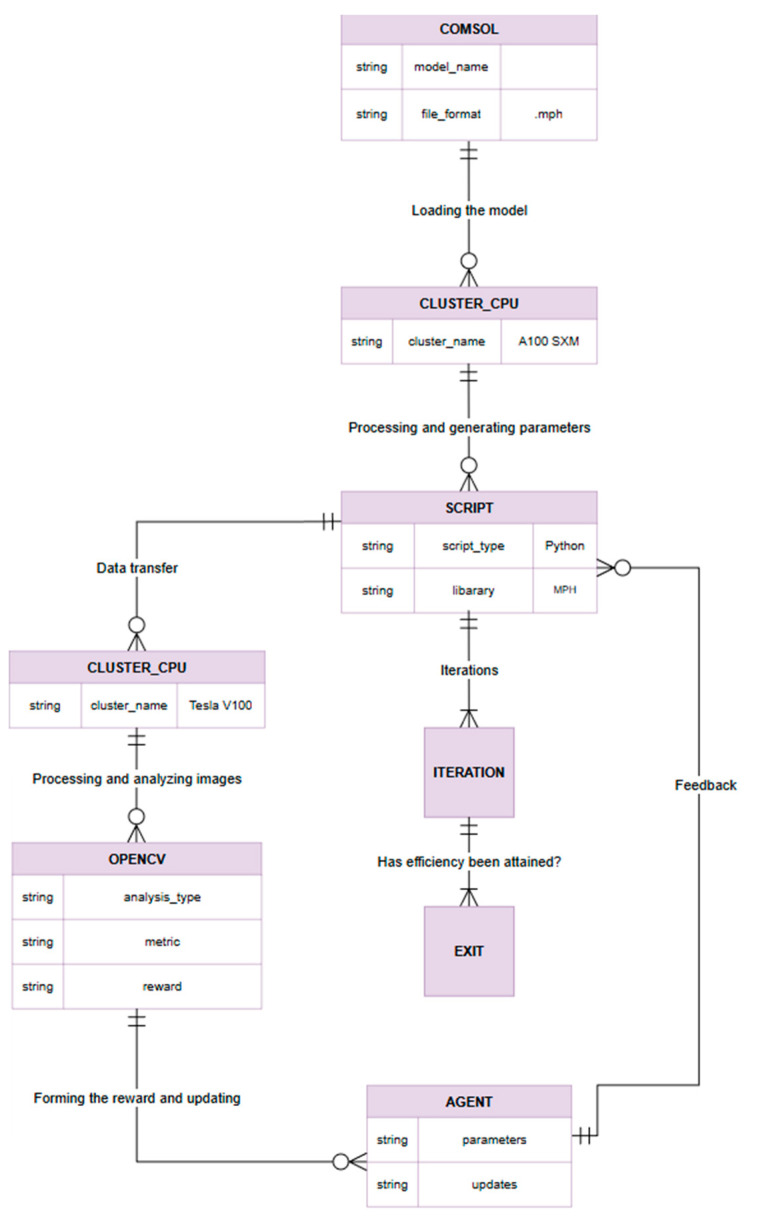
ER diagram that displays the main entities and their relationships.

The integrated simulation–learning workflow proceeds as follows (Figure 5):

1. Model Initialization: A detailed COMSOL model of the microfluidic device is constructed with specified initial input parameters (e.g., fluid flow rates, acoustic wave frequency), establishing the baseline configuration for simulation.
2. HPC Deployment: The COMSOL model is deployed and executed on a high-performance computing resource (the Dardel CPU cluster) to handle the computationally intensive simulations efficiently.
3. Automated Parameter Control: A Python control script (using the COMSOL–Python LiveLink via MPh) loads the model and programmatically varies the input parameters to explore different operating conditions. Each new parameter set is fed into the COMSOL solver automatically.
4. Data Transfer for Analysis: The simulation outputs—including any generated data or images of particle distributions—are collected and transferred to a GPU-accelerated environment (the Alvis GPU cluster) for rapid data processing.
5. Performance Evaluation: A computer vision module (built with OpenCV) analyzes the COMSOL output to extract performance metrics (for instance, the degree of particle separation or sorting efficiency). From these metrics, a quantitative reward value is computed, reflecting how well the current simulation met the desired objectives.
6. Agent Update: The reinforcement learning agent receives the computed reward (and relevant state data) and uses this feedback to update its policy. Based on the updated policy, the agent selects a new set of control parameters for the next experiment (i.e., it generates the next candidate input values to test on the COMSOL model).
7. Iterative Learning Cycle: Steps 3–6 are repeated iteratively, forming a closed learning loop. With each cycle, the agent refines the control strategy and the COMSOL simulation moves closer to the optimal separation performance. This iterative process continues until the microfluidic system achieves the targeted particle separation criteria or other convergence conditions are satisfied.

By integrating COMSOL and Python in this manner, the framework facilitates an autonomous, iterative learning cycle for microfluidic optimization, harnessing the accuracy of numerical simulations and the adaptability of machine learning in tandem. The approach leverages prior findings that even failure outcomes can inform and accelerate learning in microfluidic control tasks, ultimately reducing the time and experiments needed to identify optimal operating parameters.

### 3.2. Learning Process

In the article [59], it was shown that failure results can be used for microfluidic control in reinforcement learning (RL). It is expected that with this approach, RL can replace the methods required for precise control of the device and significantly reduce the time needed to select the parameters necessary for efficient particle separation. Although RL requires large data sets, it is suitable for microfluidic experiments because large data sets can be obtained by capturing video of microparticle movement through the microchannel system and splitting them into still images. The input parameters were chosen as flow rates (controlled by the pump pressure). As the velocity reference values approach the optimal separation conditions, more particles will pass through two of the three output channels, depending on the device design. It is assumed that results close to correct separation (although still failures) are key to improving the efficiency of learning and inference; therefore, gradient rewards are defined to account for the degree of failure. To assess the effect of reward settings, the number of trials taken while the two types of particles (large/small) were completely separated was compared. The initial velocity reference values were chosen as the starting point.

**Figure 6:**
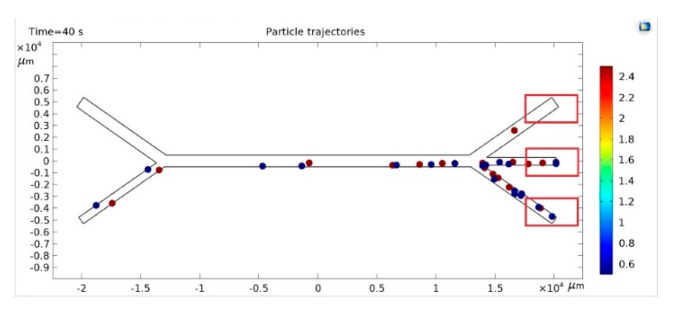
Distribution of particles in microchannels.

Water with microparticles was injected into the lower left orifice. At the same time, pure water was injected into the upper left orifice. The number of particles was counted after the injected suspension containing small and large particles reached the outlets (Figure 6). These data were used for training and inference for lobe sorting.

The physical characteristics of the particles (densities and radius), initial velocities on the input channels, and the frequency of the acoustic wave were entered into the model. The motion of a fixed number of particles (20 particles of each type) in 0.1 s steps lasting from 60 to 120 s was recorded as a video. 105 experiments were conducted for a 1 mm wide channel. The resulting video sequences were segmented into still images using image processing software.

The Open-Source Computer Vision Library (OpenCV) (https://opencv.org/) was used for training and inference, followed by ML TensorFlow (https://www.tensorflow.org/). The entire learning process is shown in Figure 6. In the machine learning process, the launch of new reference values and the observation process in the microchannel are replaced by the test data set that has already been collected. The study of the particle distribution in the three microchannels, which is time-consuming, is replaced by machine learning. Failed experiments are also involved in improving the learning process by establishing gradient rewards weighted by the degree of failure and provide an understanding of the criteria for maximizing particle sorting efficiency in microfluidic experiments.

**Figure 7:**
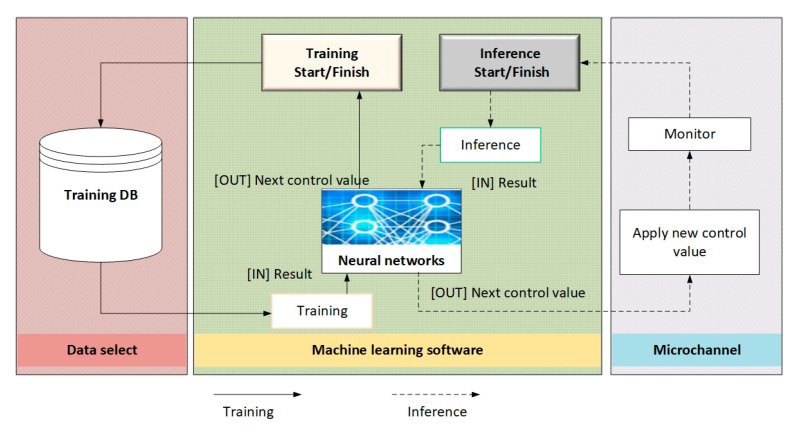
Summary of the learning process.

Figure 7 summarizes the learning and prediction process using a neural network to train the inference and distribution of particles in microchannels.

Left side (red block)—Training database. First, the necessary input data are selected (for example, particle characteristics—density, radius, initial velocities, frequency of acoustic waves, etc.). The data from the database that will be used to train the neural network.

Central part (green block)—“Neural networks”. In this block, training (training) and inference (prediction) are performed using the selected ML platform (in particular, TensorFlow).

There are separate startup parameters: “Start/Stop training” initiates or stops the training process and “Start/Stop inference” initiates or stops the prediction process.

After processing the input data (images or other parameters), the network returns the “Training result” (for internal analysis, in particular errors or accuracy) and “Next control values” (i.e., parameters that can then be used in the experiment).

During the experiment, “starting new control values” or “observation process in the microchannel” can be replaced by test data that has already been recorded.

Right side (blue block)—“Microchannels and results analysis”

Here, the analysis of the inference results takes place, i.e., an assessment of how correctly the network predicted the movement and arrangement of the particles. Based on this analysis, the “start of a new reference value” is created—i.e., the parameters of the acoustic wave, flow rate or other conditions in the microchannel can be changed for the next experiment or verification cycle.

The arrows between the blocks show the flow of data from the stage of collecting and preparing information (videos, images) by the neural networks to the final analysis and returning the control signal to the experiment.

### 3.3. Training Process

**Figure 8:**
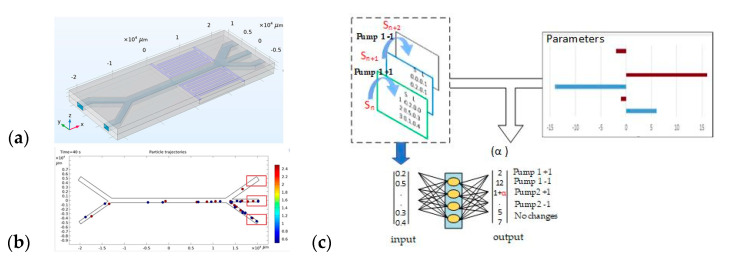
Particle sorting training process: (**a**)—general view of the microfluidic device, (**b**)—analysis of small and large particle distribution in the outlet channels, (**c**)—AI studies the relationship between input and output data.

This cyclic connection allows the model to be improved over time, including through failed experiments: they add information about the conditions under which the model may be incorrect. The process of learning to sort particles is shown in Figure 8.

Figure 8 shows the basic experimental approach and the method of applying reinforcement learning to sort particles in an acoustic-fluid system. Figure 8a, general view of the microfluidic device is shown. In the second half of the horizontal channel, an acoustic force starts acting on the particles, which decides on the separation of particles according to their physical properties (size and density).

Figure 8b, pump_1_ supplies a suspension with microparticles to the first channel, and pump_2_ supplies purified water to the second channel. While passing through the area of application of the acoustic force, smaller particles continue to move near the channel wall, and larger ones concentrate in the area of the zero acoustic pressure region. Then, the particles pass through one of the three exit channels and count the number of appropriately sized particles that have fallen into each of the exit channels.

Figure 8c shows the scheme of artificial intelligence and reinforcement learning. This figure shows the logic of the interaction between the “input state” (S_n_) and the “output state” (S_n+1_) via a neural network.

The state S_n_ contains information about the current settings of the fluid flow (pumps 1 and 2), the frequency of acoustic waves, and data about the physical properties and distribution of particles. Based on this, the RL agent selects an action, i.e., a way to change the flows or frequency of the acoustic wave.

After the action is completed, a new state S_n+1_ appears and a reward α, which measures the success of the sorting (in particular, the number of large and small particles that hit the target zones). This “reward” is added to the neural network together with the action and states, so that the algorithm can learn and refine the choice of actions in the next steps.

To ensure the physical consistency of the reinforcement learning process in COMSOL-based simulations, the reward was formulated as a normalized, multi-objective function:

(6) $$R=1-{Frac}_{wrong}-\lambda_{fail}C_{phys}-\lambda_{energy}\frac{∆P}{∆P_{max}}$$

where ${Frac}_{wrong}$ the fraction of incorrectly sorted particles per episode (number of particles outside the target outlet divided by the total particle count), normalized to [0,1].

ω(fail) a weighting function for failed experiments (0 = near-correct separation; 1 = complete failure such as full stream overlap), combined with the coefficient $\lambda_{fail}$.

$C_{phys}$—aggregated penalty for physical or technological constraint violations, $C_{phys}=min(1,\sum_{k}c_{k})$, where $c_{k}$ are normalized individual violations (e.g., acoustic frequency or inlet velocity exceeding stability limits).

∆P**/**$∆P_{max}$—normalized energy-efficiency term; ∆P represents either the acoustic pressure amplitude in the SAW region or the hydrodynamic pressure drop between inlets and outlets, and $∆P_{max}$ is the maximum permissible value ensuring wave stability and avoiding cavitation or overheating.

The coefficients $\lambda_{\mathrm{fail}}$, $\lambda_{\mathrm{phys}}$, and $\lambda_{\mathrm{energy}}$ (typically 0.1–0.5) were optimized empirically to balance convergence speed, physical stability, and energy efficiency.

This normalized reward formulation allows stable PPO training and can be easily extended to other COMSOL-based learning tasks involving thermal, mechanical, or electromagnetic domains.

The “reward” is calculated as the sum of the products of the “particle parts” of a certain size that ended up in each output channel, using the appropriate coefficients (parameters):

(7) $$\alpha =\sum_{i=1}^{3}\sum_{j=1}^{2}K_{i,j}^{\left(n+1\right)}\times P_{i,j}$$

where i—index of the output channel (i=1, 2, 3), j—index of the particle type (j=1 for large particles, j=2 for small particles), $K_{i,j}^{\left(n+1\right)}$—fraction of particles of type j that reached channel i at iteration n+1 (normalized to the total number of injected particles), and $P_{i,j}$—weighting parameter (positive for “desired” combinations and negative or zero for “undesired” ones).

Thus, the higher the fraction of correctly sorted particles in their respective channels, the greater the total reward value. This formulation enables the reinforcement learning agent to maximize sorting efficiency while penalizing misclassifications.

Equation (8) describes how the “***Output column***” for each possible action is created, based on the current state Sn and α of the reward:

(8) Output column=(result of inserting S_n into NM)+reward a

First, a state Sn (which indicates in particular the distribution of the particles) is fed into the input of the neural network. The neural network computes numerical values (the “output”) for all possible actions that can be performed from this state. The reward is α calculated separately (Equation (7)) based on the ratio of particles of different sizes for each position. During training, the reward α is added only in the row of the output column that corresponds to the performed action (i.e., the transition from state S_n_ to S_n+1_).

Therefore, the “***Output column***” actually combines the previous predictions of the neural network about the utility of each action with the current “reward” α. This allows the reinforcement learning algorithm to select actions with the highest overall value and, as a result, better learn how to control the pump speed to achieve the target particle distribution.

The reference values here were the changes in the amount of water in each pump. “Pump 1+1” meant that the speed should be increased by 1.0 μL/min or left at 4.0 μL/min if it was already at maximum speed. “Pump 1−1” meant that the speed should be decreased by 1.0 μL/min or maintained at 1.0 μL/min if it was already at the lowest speed. The situations with “Pump 2+1” and “Pump 2−1” were the same as for pump 1. “No change” meant that the reference value should not change.

The particles were controlled by changing the acoustic wave frequency and the fluid flow rate. The acoustic wave frequency was selected depending on the microchannel width, and the velocity varied from 0.3 mm/s to 2 mm/s at the entrance to channel 2 and from 0.6 mm/s to 6 mm/s at the entrance to channel 1.

**Figure 9:**
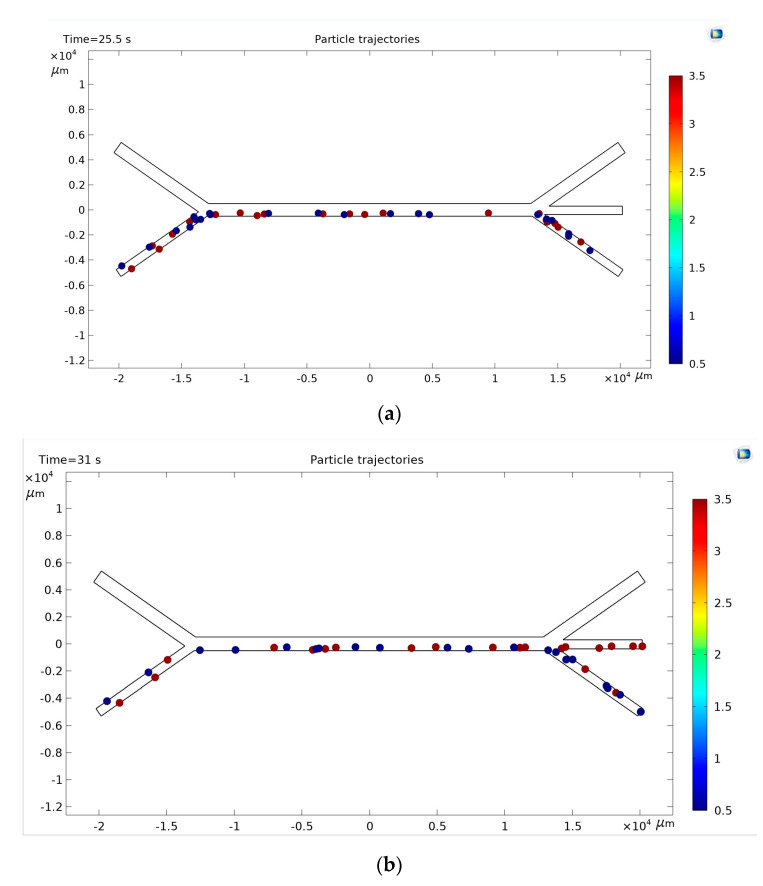
Distribution of microparticles in channels: (**a**)—without the application of acoustic force, (**b**)—in the presence of acoustic force.

The parameter settings for calculating the rewards reflecting the failure results are shown in Figure 9 and Table 2.

The particles reached a specific exit channel depending on their size, flow rate and device design. Without the use of acoustic force, all particles fell into channel 3 (Figure 9a). In the presence of acoustic force, small particles fell into channel 3 or channel 2, and larger particles into channel 2 or channel 1 or 3 (Figure 9b).

Therefore, channel 3 is considered as the target region for smaller particles, and channel 2 is considered as the target region for larger particles. It was expected that more particles would be collected in the target regions when the reference values approached the optimal separation conditions. Therefore, it was hypothesized that results close to the target region (still failure results) are the key to improving the efficiency of learning and inference in ML.

Experiments were analyzed in which the particle sorting was performed with errors (for example, large particles entered the “wrong” zone). These unsuccessful experiments allowed us to adjust the “reward parameters” so that the reinforcement learning algorithm would have a clearer error signal.

**Figure 10:**
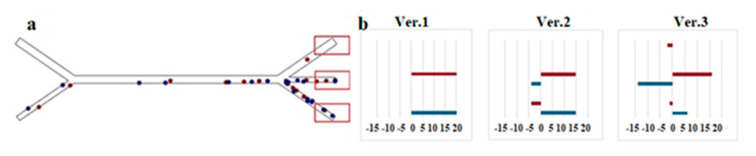
Using failure results to calculate reward. (**a**) Target regions reached by the particles depending on the shape of the microchannel. It was expected that the separation results would be concentrated around the target region as the sorting process was completed. (**b**) Parameter settings characterized by using the failed sorting results to find the corresponding condition.

Figure 10b shows several setting options, each of which shows exactly how to take into account “bad” (negative) or “good” (plus) sorting results. In particular, case 3 (when particles do not enter their target channels, negative coefficients are assumed to emphasize the difference) is proposed to display the damage results. For comparison, case 1 (particles entering only the target channels) and case 2 (particles entering the target channels most often) are also included. The idea is that during training, the neural network “learns” to avoid situations that caused the misclassification of particles.

As can be seen from Table 2, the parameter values were distributed among the three channels in such a way that the absolute value was equal to 3. Since the exit channels through which the particles pass differ depending on their size, different parameters were set for each case.

## 4. Results

**Figure 11:**
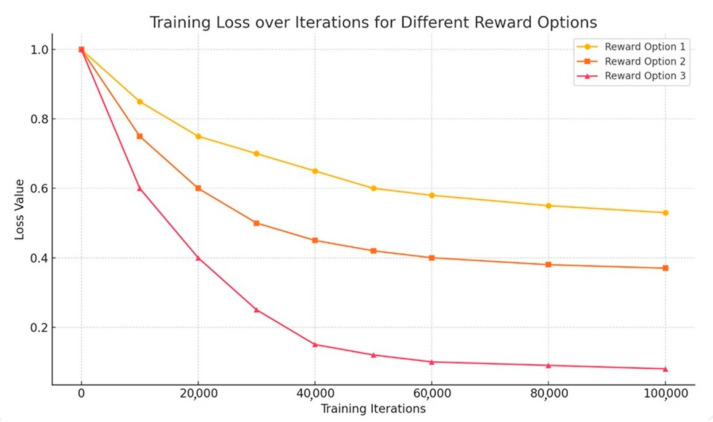
Training results.

The results of training are presented in Figure 11. During the preparation of the training data, the control conditions were adjusted as follows: the fluid flow rate provided by pump 1 was set to 6, 5, 4, 3, 2, 1.5, 1, 0.8, 0.7, and 0.6 mm/s, while pump 2 operated at 0.7, 0.6, 0.5, 0.4, and 0.3 mm/s. The number of training data is the total number of pairs randomly extracted from 45,000 (10 × 5 × 15 × 60) results of multiple division with the possibility of duplication. The loss was calculated by the method of least squares in the replay of the experience. In the reward set in case 3, the loss was kept low after training with 10 × 105 data, and effective training was achieved. This was achieved because all separation data, including data in channels other than the target ones, were used for training, rewards were set low with minus values for data prone to producing failure results, and rewards were high when divided successfully.

Figure 11 demonstrates the results of training the machine learning model used in the sorting process in a microfluidic system. The ***x***-axis depicts the number of learning iterations (up to 100,000), and the ***y***-axis shows the loss values. The loss function measures how accurately the model predicts sorting results. The lower the losses, the more accurately the model works. Losses are used as a feedback mechanism to optimize model parameters. Reduced losses means that the model better predicts sorting results.

A graph illustrating the change in the value of the loss function during training for three variants of the reward system: Reward Option 3 shows the fastest and most stable loss reduction—the most effective training; Reward Option 2 has moderate efficiency, and Reward Option 1 learns more slowly and less steadily. The lower losses in Case 3 emphasize that the model trained with this setting is quite accurate and efficient for sorting microlobes.

For clarity, the median sorting error reported in this section corresponds to ${Frac}_{\mathrm{wrong}}$ in the reward definition (median = 0.041, IQR 0.032–0.052).

The normalization of the reward components ensured comparable scales between the physical and learning domains, allowing efficient convergence across multiple COMSOL simulation conditions.

**Figure 12:**
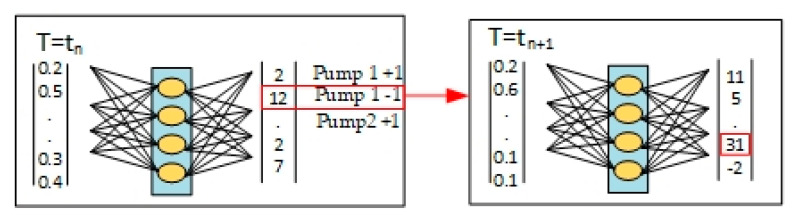
Iterative process of automated microchannel control for sorting particles using artificial intelligence.

The performance of the trained AI was validated using a different data set (smaller particles had a density of 1050 kg/m^3^ and a radius of 0.5 μm, and large ones had a density of 5250 kg/m^3^ and a radius of 3.5 μm, respectively), obtained in the same way as the training data. Sorting data was entered into the trained AI, and values for each control condition were output, as shown in Figure 12. For each parameter (Option 1, Option 2 and Option 3), the number of training data was recorded at the level of 10 × 105. The microchannel was manipulated according to the highest score in the output column, and this process was repeated until the desired separation results were obtained.

At each step, T = ***t***_n_ + 1. The system collects input data (sorting results, i.e., sorted or unsorted particles, microchannel status, etc.). This input is fed to the input of the neural network. According to the received input values, it determines the output signals—in the example in the figure, this is the value for Pump 1+1, Pump 1–1 or Pump 2+1, Pump 2−1. That is, the neural network “decides” how to control the pumps in the next step in order to better sort the particles, and “+1” or “–1” mean different modes of operation of the pump. For example, let us consider an example, “Pump 1−1”, when it is turned on in a real installation. As a result, the microchannel enters a new state T = ***t***_n_ + 1, after which the system measures the sorting result again and repeats the cycle.

In this way, the algorithm controls the device in several steps, each time choosing the best control signal (pump or its speed) emitted by the neural network. Iterations continue until the desired particle sorting indicator is achieved or improved (e.g., minimizing residual particles in the “wrong” channel).

The study demonstrated the effectiveness of using a trained neural network model to predict and control the distribution of microparticles based on various input parameters, such as microparticle size, density, flow rate, and acoustic pressure. The model successfully predicted sorting results, demonstrating its potential for application in a real-world process.

## 5. Discussion

The results confirm the effectiveness of a closed-loop control system based on reinforcement learning for sorting microparticles in acoustofluidic devices. The reward configuration that includes penalties for incorrect sorting (Option 3) significantly improved model performance by accelerating convergence and reducing training loss. This indicates that failure data, when properly weighted, can be a valuable source of information for learning algorithms.

The cyclic interaction between the AI model and the physical system allowed the neural network to iteratively refine its control strategy. By analyzing the current state (particle distribution, acoustic parameters, flow rates), the agent selected optimal actions that guided the system toward the desired separation outcome.

This control loop mimics real-time feedback operation and is scalable to more complex microfluidic architectures. The model successfully predicted the movement and sorting of particles based on their physical characteristics and channel conditions. These findings support the feasibility of applying reinforcement learning to the autonomous control of lab-on-a-chip devices in real experimental setups.

The presented approach could significantly reduce the need for manual parameter tuning and experimentation time, thereby accelerating the design cycle and deployment of acoustofluidic systems. To strengthen the focus and highlight the immediate significance of our acoustic-microfluidic approach, articles should concentrate on two key application scenarios: environmental monitoring, focusing on the separation and analysis of microplastics and soot/ash particles in fluid samples, a direction strongly supported by our current theoretical model, and validation using polyethylene microspheres.

To validate the results obtained from numerical modeling and AI-based control strategies, experimental prototyping of the acoustofluidic Lab-on-Chip is planned. A microchannel structure will be fabricated using laser micromachining of a silicon substrate and integrated with a piezoelectric LiNbO_3_ layer and interdigital transducers (IDTs) formed via screen printing, as previously demonstrated [58,60]. The developed chip will be tested using microparticle suspensions of known properties, matching those used in simulation, under dynamically controlled flow conditions.

These experiments will aim to replicate the acoustic field configurations predicted by the COMSOL-Python integrated model and assess sorting efficiency in real-time. Bonding techniques such as double-sided adhesive film will be applied to ensure stable and leak-free microchannel sealing, enabling repeatable fluidic experiments [58,60]. Real-world data obtained during these tests will serve both to evaluate the model’s predictive accuracy and to refine reinforcement learning algorithms used in control signal selection.

## 6. Conclusions

This article presents the results of Microparticle Separation Efficiency in Acoustofluidic Chips via Machine Learning and numerical modeling, which can be applied to environmental monitoring, specifically for detecting the content of microplastics in water. The authors plan to conduct an experimental study in a clinical liquid in the future. This paper discusses the separation model of two types of suspended particles of a mixture dispersed in a branched channel under the influence of an acoustic field for the purpose of optimal selection of input parameters. It is shown that determining the frequency of acoustic waves is possible using regression analysis. The regression equations for ***f_min_*** and ***f_max_*** showed an average approximation error of 1.71% and 6.47%, respectively, which suggests their statistical reliability.

To determine the fluid input velocities, a machine learning with reinforcement by failures was used. This algorithm demonstrates the application of machine learning to improve accuracy and efficiency in model studies and under experimental conditions, providing a new perspective on the problem of separation of biological materials in laboratory chips with acoustic fluids.

Based on numerical experiments, a data set was prepared for training the algorithm. Information on the physical parameters of particles, flow rate, and acoustic interaction frequency was used.

A reinforcement learning algorithm was implemented, which allows for the optimization of microparticle sorting parameters. A feature of the algorithm is the inclusion of failure results, which contributes to a faster and more accurate learning process.

The algorithm was tested on new data sets, which confirmed its effectiveness in predicting optimal parameters and increasing the accuracy of particle sorting.

The developed algorithm ensures the unification of the microfluidic system design process. This allows for reducing the time and resources spent on creating new microfluidic devices, increasing their efficiency, and facilitating the integration of such systems with various industries, for instance in clinical diagnostics through lab-on-a-chip devices for rapid blood or exosome analysis, in environmental monitoring for automated microplastic and pollutant detection in water samples, in the pharmaceutical sector via organ-on-a-chip platforms for drug screening and toxicity testing, and in the food industry for quality control and pathogen detection.

## Figures and Tables

**Table 1:** Choice of velocity options with 100% separation of two particle types.

*ρ*_1_, kg/m^3^	*ρ*_2_, kg/m^3^	R1, M	R2, m	V1, m/s	V2, m/s
1050	10,500	0.5 × 10^−6^	10 × 10^−6^	0.0007	0.0003
1050	10,500	0.5 × 10^−6^	5 × 10^−6^	0.0020	0.0005
1050	10,500	0.5 × 10^−6^	5 × 10^−6^	0.0012	0.0004
1050	10,500	0.5 × 10^−6^	5 × 10^−6^	0.0009	0.0003
1050	5520	0.5 × 10^−6^	5 × 10^−6^	0.0010	0.0003

**Table 2:** Setting the parameters for calculating the reward.

Parameter Set	Particle Size	Target Area		
		Channel 1	Channel 1	Channel 1
Version 1	Large (red)	0	3	0
	Small (blue)	0	0	3
Version 2	Large (red)	0	2	1
	Small (blue)	0	1	2
Version 3	Large (red)	−1	1	−1
	Small (blue)	−1	−1	1

## Data Availability

The original contributions presented in this study are included in the article. Further inquiries can be directed to the corresponding author.
